# The role and metabolic adaptations of neutrophils in premetastatic niches

**DOI:** 10.1186/s40364-023-00493-6

**Published:** 2023-05-09

**Authors:** Enli Chen, Jing Yu

**Affiliations:** Cancer Center, Beijing Friendship Hospital, Capital Medical University, No. 95 Yong an Road, Beijing, 100053 Xi Cheng District China

**Keywords:** Neutrophil, Cancer, Pre-metastatic niches, Metastasis, Metabolism

## Abstract

It has been found that tumor cells create microenvironments in distant organs that promote their survival and growth in advance of their arrival. These predetermined microenvironments are referred to as “pre-metastatic niches”. Increasing attention is being paid to neutrophils’ role in forming the pre-metastatic niche. As major components of the pre-metastatic niche, tumor-associated neutrophils (TANs) play an important role in the formation of the pre-metastatic niche through communication with multiple growth factors, chemokines, inflammatory factors, and other immune cells, which together create a pre-metastatic niche well suited for tumor cell seeding and growth. However, how TANs modulate their metabolism to survive and exert their functions in the process of metastasis remains largely to be discovered. Accordingly, the objective of this review is to assess the role that neutrophils play in the formation of pre-metastatic niche and to explore the metabolism alteration of neutrophils in cancer metastasis. A better understanding of the role of TANs in pre-metastatic niche will help us discover new mechanisms of metastasis and develop new therapies targeting TANs.

## Background

Metastasis formation requires not only a specific genetic profile of tumor cells that enhances metastatic ability but also a modified local microenvironment at distant sites. As a result of tumor-induced changes, tissues are more receptive to tumor cells dispersed throughout the body. Factors derived from the primary tumor are responsible for inducing "pre-metastatic niche" formation before tumor cells reach the target organ. In 2005, this novel concept was the first proposal by Kaplan et al. [1]. Their study showed that hematopoietic progenitor cells from bone marrow expressing vascular endothelial growth factor receptor 1 (VEGFR1) accumulated early in premetastatic lungs, and mice were prevented from forming pre-metastatic niche when VEGFR1 function was blocked by antibodies or by removing VEGFR1 positive cells from their bone marrow [1].

Several studies have demonstrated that more neutrophil infiltration of tumors is associated with worse clinical prognosis [2–4]. However, the exact role of neutrophils in the formation of pre-metastatic niche remains unknown. Besides, to maintain their effector function, neutrophils must consume a lot of energy and adapt quickly to their metabolic environment. Despite recent interest in immunometabolic research in cancer, researchers have focused on how metabolic changes affect the function of T cells. The metabolism of neutrophils in pre-metastatic niche is still poorly understood. Here, we review the current evidence regarding neutrophils' role and metabolic adaptations in pre-metastatic niche formation.

## Tumor-derived factors in recruiting neutrophils

**Fig. 1:**
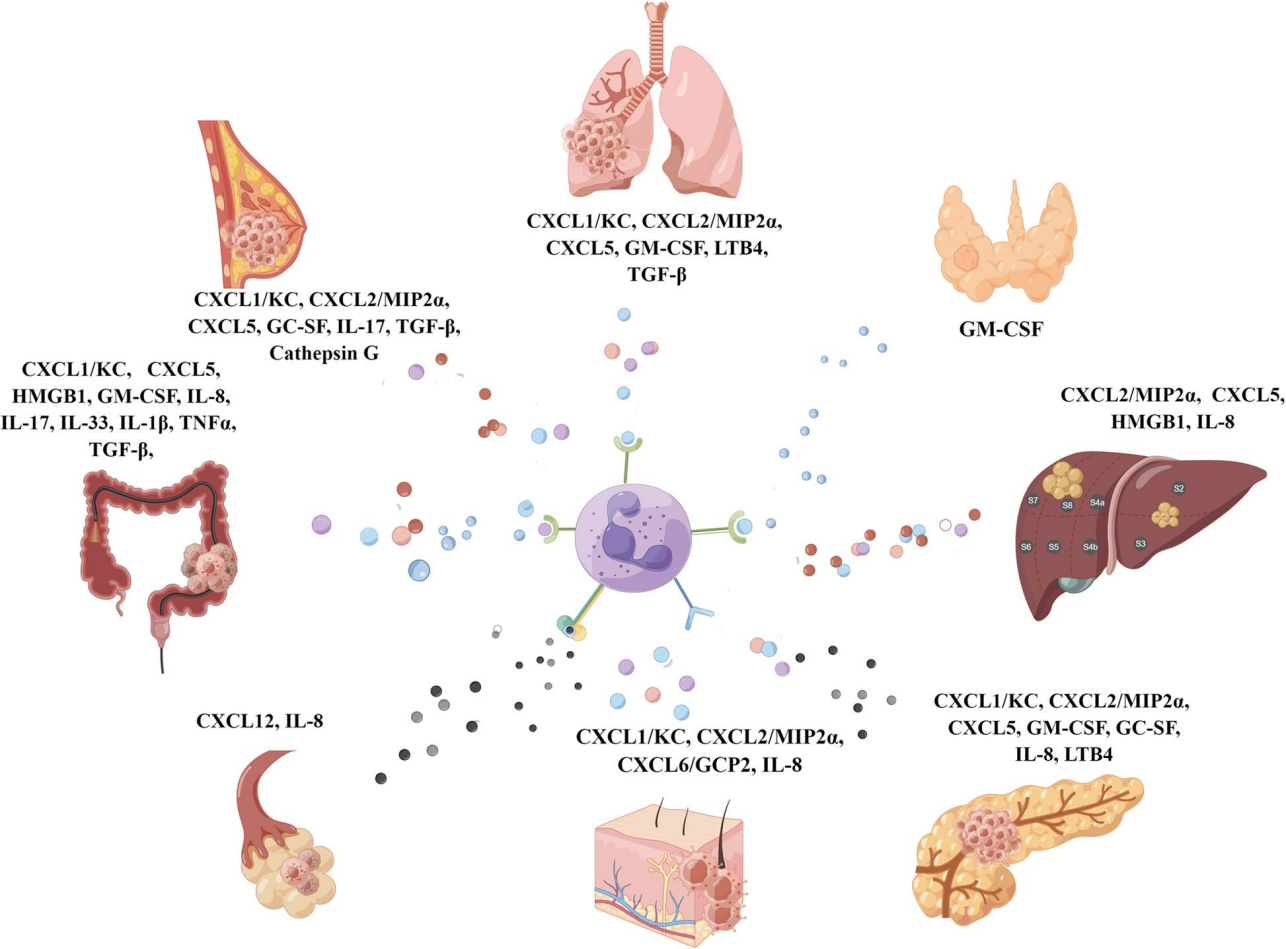
Different tumor-derived factors in recruiting neutrophils. Different types of tumor cells characteristically secrete a range of chemokines and inflammatory factors to promote neutrophil infiltration. CXCL: C-X-C motif chemokine ligand; MIP2 α: macrophage inflammatory protein-2 alpha; IL: interleukin; HMGB1: high-mobility group box 1; GM-CSF: granulocyte–macrophage colony-stimulating factor; G-CSF: granulocyte colony-stimulating factor; TNF- α: tumor necrosis factor-alpha; LTB4: leukotriene B4; GCP-2: granulocyte chemotactic protein-2

Many studies have demonstrated that tumor-derived factors are responsible for recruiting and activating tumor-associated neutrophils (TANs). G-CSF is the most important signal for granulopoiesis. The secretion of G-CSF promotes the differentiation of hematopoietic cells into neutrophils. Moreover, G-CSF stimulates neutrophil recruitment to tumors. Breast cancer, pancreatic cancer, and lung cancer cells can secret G-CSF to attract neutrophils expressing G-CSFR [5–7]. Colorectal cancer, pancreatic cancer, lung cancer, and thyroid cancer cells can produce a large amount of GM-CSF to attract GM-CSFR positive neutrophils [5, 8, 9]. In hepatocellular carcinoma, ovarian cancer, melanoma, and pancreatic cancers, tumor cells can release IL-8, which can attract CXC chemokine receptor 1/2 (CXCR1/2) positive neutrophils [10, 11]. Breast cancer cells secret IL-17 and TGF-β that attract neutrophils expressing IL-17R/ TGF-βR [12, 13]. Xiao et al. showed that tumor-secreted protease cathepsin C (CTSC) activates neutrophil membrane-bound proteinase 3 (PR3) to promote IL-1β processing and NFκB activation, finally upregulating IL-6 and CCL3 for recruitment of neutrophils [13]. Furthermore, colorectal cancer cells are capable of producing IL-17, IL-33, TNF-α, and TGF-β to guide IL-17R/ST2/ TNF-αR/ TGF-βR positive neutrophils directional migration [14–17] (see Fig. 1 for details). Satpathy et al. found that crystalline silica (CS)-induced neutrophil recruitment is dependent on LTB4 production by mast cells and BLT1 expression on neutrophils. Then they used an implantable lung tumor model and indicated that CS exposure resulted in rapid tumor growth and decreased survival that could be attenuated in the absence of BLT1 [18]. It has also been shown that CXCL16 and its receptor CXCR6 play an important role in the recruitment of neutrophils in hepatocellular carcinoma [19]. Besides, a variety of cancers have heightened levels of neutrophil-specific chemokines such as CXCL6, CXCL8 (IL-8), and CCL3 (MIP-1α) [20, 21]. A high percentage of infiltrating TANs has been revealed to be associated with an advance in the formation of pre-metastatic niche. It has been found that TANs contribute significantly to tumor metastatic colonization, facilitating pre-metastatic niche formation, and promoting immunosuppression through their various mechanisms. In conclusion, tumor cells secret a spectrum of chemokines, cytokines, and growth factors to attract TANs. However, more research is still needed to explore the mechanism of secretion of these tumor-derived factors.

## Neutrophil chemotaxis towards pre-metastatic niche

### Effect of chemotactic factors on TANs migration towards pre-metastatic niche

Chemotactic factors play a significant role in neutrophil migration. In terms of potency, the CXCL family of chemokines is the most effective. Among them, the neutrophil chemokine axis CXCR4/CXCL12 has received the most attention. It is noteworthy that CXCL12 levels are elevated in pre-metastatic niche including the liver, bone, lungs, brain, and lymph nodes. Muller et al. found that when CXCR4/CXCL12 interactions were inhibited in vivo, breast cancer metastasis was significantly reduced [22]. Yu et al. showed that through CXCR2-dependent recruitment of neutrophils, TNF-α-activated mesenchymal stromal cells promoted breast cancer metastases. Conversely, deficiency of CXCR2 inhibited neutrophil infiltration in murine breast cancer models and inhibited the occurrence of metastases [23]. Soler-Cardona et al. demonstrated that a melanoma cell line overexpressing CXCL5 could recruit more neutrophils to lymph nodes and boost lymph node metastasis [24]. Previous studies on lewis lung carcinoma mouse models showed that blockade of chemokine receptor CXCR2 or CXCR2-deficient mice reduced angiogenesis and tumor growth [25]. Tissue inhibitor of metallopeptidase 1 (TIMP1), an enzyme involved in extracellular matrix (ECM) remodeling, can promote liver pre-metastatic niches formation by recruiting neutrophils through stromal cell-derived factor 1 (SDF1/ CXCL12)-CXCR4 dependent way [26]. The chemokine CCL2 is released by tumors in large quantities and attracts CCR2-positive neutrophils into pre-metastatic niche [27].

### Effect of extracellular vesicle (EV) and exosome from tumors on neutrophil migration towards pre-metastatic niche

According to recent studies, EV and exosomes released by tumor cells contribute to pre-metastatic niche formation, promoting metastasis. Exosome is an extracellular vesicle that ranges in size from 30 to 200 nm, released by all cells. By transferring information via their cargo, including proteins, DNAs, RNAs, and microRNAs, EVs, and exosome play a crucial role in intercellular communication between cancer cells and pre-metastatic niche through blood circulation. In a recent study, toll-like receptor 3 (TLR3) on lung epithelial cells is responsible for triggering neutrophil recruitment and lung pre-metastatic niches by sensing tumor exosomal RNA to induce secretion of chemokines (CXCL1, CXCL2, CXCL5, and CXCL12) [28]. According to Maximiliane et al., melanoma cells secrete a distinct EV subtype when the Bcl2-associated anthogene 6 (BAG6) protein and p53 acetylation are disrupted, which forms a distant pre-metastatic niche. The formation of anti-tumor-EVs was dependent on acetylation of p53 by the BAG6/CBP/p300-acetylase complex, followed by recruitment of components of the endosomal sorting complexes required for transport (ESCRT) via a P(S/T)AP double motif of BAG6. When BAG6 was ablated and this pathway was disrupted, a distinct EV subtype was released, recruiting tumor-promoting neutrophils to the pre-metastatic niche, resulting in the progression of tumors and metastasis [29]. Besides, in a triple-negative breast cancer model, Qi et al. demonstrated that LIN28B released by tumor cells attracted neutrophils and established an immunosuppressive pre-metastatic niche. As a result of LIN28B stimulation, neutrophil N2 conversion in lung pre-metastatic niche was enhanced. By up-regulating PD-L2 and dysregulating cytokine levels, N2 neutrophils function to suppress immune function in the pre-metastatic lung. In addition, they found that exosomes released by breast cancers with low Let-7 s played an important role in neutrophil recruitment and LIN28B-induced formation of pulmonary pre-metastatic niches. There is further evidence that both high Lin28B and low Let-7 s are associated with poor prognosis and lung metastases in patients with breast cancer [30].

### Other bioactive factors

The migration of neutrophils is also regulated by myeloid-related proteins (MRPs). There is a strong expression of MRPs in the pre-metastatic niche, such as S100A8 or S100A9, which act as potent neutrophil chemoattractants [31]. In the angiogenic environments of malignant glial tumors, hypoxia-inducible factor 1α (HIF1α) and its products such as CXCL12, VEGF, or MMP9 also play a role in neutrophil recruitment and retention [32]. In addition, VEGF increases neutrophil adhesion to postcapillary venules, which promotes the efficient homing of neutrophils to tissues expressing high levels of VEGF [33]. Besides, complement component 3 (C3) is produced by lung mesenchymal stromal cells (LMSCs) in response to Th2-type cytokines. This leads to the recruitment of neutrophils with high levels of C3a receptors to pre-metastatic niche, supporting metastasis [34]. Huang et al. identified a group of lymphatic endothelial cells (EC) with increased Ki67 and S100A6 expression,which impaired EC tight junction and increased the transendothelial migration of neutrophils [35].

## Change of neutrophils’ polarization state in pre-metastatic niche

**Fig. 2:**
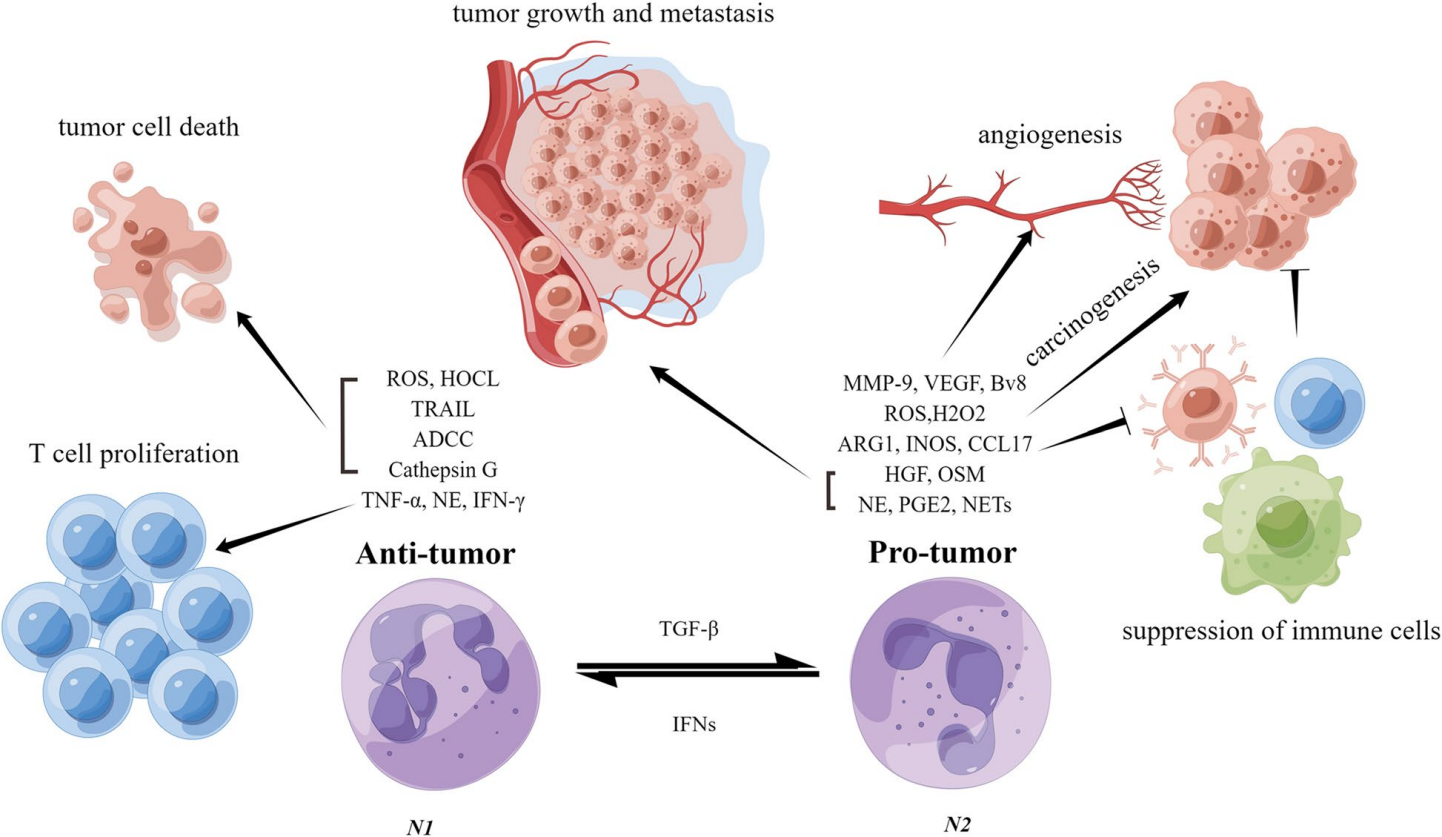
The differentiation and role of TANs in pre-metastatic niche. IFNs can foster antitumor phenotype (N1), whereas transforming growth factor-β (TGF-β) can promote pro-tumor phenotype (N2). N1 exerts anticancer effects via different mechanisms of action like reactive oxygen species (ROS), hypochlorous acid (HOCL), TNF-related apoptosis-inducing ligand (TRAIL), antibody-dependent cell-mediated cytotoxicity (ADCC), cathepsin G, tumor necrosis factor-α (TNF-α), Norepinephrine (NE), and Interferon-γ (IFN-γ). N2 exerts a cancer-promoting effect by promoting tumor angiogenesis, growth, and metastasis, and suppressing the activation of immune cells. Matrix metalloprotein, MMP9; Vascular endothelial growth factor, VEGF; Arginase-1, ARG1; Inducible Nitric Oxide Synthase, INOS; C–C motif chemokine ligand 17, CCL17; Hepatocyte growth factor, HGF; Oncostatin M, OSM; Prostaglandin E2, PGE2; Neutrophil extracellular traps, NETs

When neutrophils accumulate in premetastatic niches, they can change their polarization state, switching from suppressive to prometastatic roles, leading to tumor metastasis [36]. CD11b + Ly6G + Ly6C + is the marker of N1 neutrophils and N1 neutrophils have cytotoxic properties caused by their production of superoxide and hydrogen peroxide, which can kill tumor cells [37, 38]. In addition, several other biological processes are involved in this process including TNF-related apoptosis inducing ligand (TRAIL), antibody-dependent cell-mediated cytotoxicity (ADCC) and Cathepsin G [39–41]. N1 neutrophils are also capable of promoting the proliferation of T cells through TNFα, NE and IFN-γ [42]. CD11b + Ly6G + Ly6C^low^ is the marker of N2 neutrophils [43]. The N2 neutrophil belongs to metastasis-promoting neutrophils, which can promote angiogenesis, tumor growth, and metastasis. Besides, N2 neutrophils can suppress the activation of various immune cells such as dendritic cells (DC), T cells, and B cells [44]. Previous studies indicate that TANs can keep functional plasticity and can undergo phenotypic change when responding to various tumor microenvironment (TME) signals like transforming growth factor TGF-β and interferon signal [45, 46]. It was indicated that type I interferons had a strong ability to kill tumor cells by regulating the function of neutrophils and promoting neutrophil-mediated antitumor immunity [47]. TGF-β, on the other hand, can polarize neutrophils into a pro-tumor N2 phenotype (see Fig. 2 for details). Besides, IL-6 and IL-10 have been reported as an N2 inducers of neutrophils by cooperating with G-CSF. Tumor cells can cause N2 transformation in pre-metastatic niche by secreting IL-6, IL-10, G-CSF, and TGFβ to pre-metastatic niche or inducing cells in pre-metastatic niche to produce these factors.

## The role of neutrophils in establishing the pre-metastatic niche

Neutrophils are recruited by multiple tumor-secreted factors, thereby establishing an immunosuppressive environment that aids the survival and metastasis of tumor cells. There has been extensive research on neutrophils among TANs with immunosuppressive functions. In TANs, immune-suppressive neutrophils are known as granulocytic myeloid-derived suppressor cells (G-MDSCs) or polymorphonuclear myeloid-derived suppressor cells (PMN-MDSCs) [48, 49]. G-MDSCs appear as neutrophils at different stages of maturation. Non-cytotoxic immunosuppressive neutrophils are immune suppressive or regulatory since they contain large amounts of Arginase 1 (Arg1) that inhibits T cell proliferation by depriving the cells of L-arginine. Mishalian et al. found that TANs can establish an immunosuppressive environment by releasing a large number of CCL17 which is a chemoattractant of regulatory T-cells (T-regs) [50]. In addition, neutrophils play an immunosuppressive role in T cell immunity partially via the expression of PD-L1 and PD-L2 [51, 52]. Previous studies have reported that there is a strong association exists between inflammation and tumor progression. However, little research has been conducted on inflammation's role in pre-metastatic niche development. A well-known fact is that during the metastatic stage, neutrophils can release proinflammatory factors such as cytokines (TNFα, IL-12) and proteases to foster pre-metastatic niche formation [53, 54]. Rayes et al. showed that bone marrow-derived neutrophils were recruited to the lungs when inflammation occurs, and neutrophils could proteolyze the anti-tumorigenic factor thrombospondin-1 (Tsp-1), finally facilitating lung metastasis [55]. Besides, the neutrophils that are mobilized by G-CSF produce Bv8 in pre-metastatic niche, a protein that stimulates angiogenesis when it is activated [56]. Neutrophils also can produce CXCL8 which is closely associated with angiogenesis. In the aspects of angiogenesis, a variety of proteases can be released by activated TANs, which degrade and remodel the extracellular matrix (ECM), assisting angiogenesis. Among neutrophil-released proteases, matrix metalloproteinases (MMPs) play a significant role in the development of tumor angiogenesis, particularly MMP-9. MMP-9 can break down ECM, releasing a variety of angiogenic factors, including VEGF and fibroblast growth factor 2 (FGF2), that act upon nearby endothelial cells to promote tumor angiogenesis [57]. Besides, many tumors express TNF-α, a cytokine that can induce neutrophil degranulation and release of VEGF, finally promoting vascular endothelial cell proliferation [58].

## Neutrophils drive the metastasis of tumors

### The role of neutrophils and its secreted factors in the metastasis of tumors

In a recent study, neutrophils were shown to tether circulating tumor cells (CTCs) to the endothelium of target organs through contact-dependent mechanisms. Neutrophil-tumor cell interactions are mediated by integrins on neutrophils binding to intercellular adhesion molecule 1 (ICAM-1) on tumor cells [58]. Huh et al. showed that the retention ability of human melanoma cells in the lungs was enhanced after injecting neutrophils into nude mice [59]. As a result of neutrophils' presence in the lungs, pro-metastatic molecules such as S100A8, S100A9, Bv8, and MMP9 are elevated, supporting tumor cell growth in the lungs [31]. Neutrophils isolated from pre-metastatic niches secrete leukotriene B4 (LTB4) that transforms heterogeneous cancer cell populations into metastasis-initiating cells and enhance the ability of metastasis through arachidonate 5-lipoxygenase (ALOX5) enzyme. Metastasis in the lungs was reduced by inhibiting ALOX5 [56, 60]. The Bv8 is also expressed in neutrophils, which facilitates the homing of tumor cells [61]. It is known that the secretion of STAT3-activated glycoprotein lipocalin-2 (LCN2) is enhanced by N2 neutrophils, which fosters the mesenchymal-to-epithelial transition (MET) process in cancer cells, restoring epithelial features of the cells, thereby facilitating colonization and metastatic outgrowth [62]. Leukotrienes generated by neutrophils appear to be effective in promoting metastatic niche formation. It has been shown that neutrophil-secreted leukotriene can promote tumor cell proliferation as well as metastatic abilities [60].

### Pro-tumor role of NETs in pre-metastatic niche

There is evidence that neutrophil extracellular traps (NETs) can act as an anti-tumor and pro-tumor agent in cancer [63]. They can kill tumors directly and prevent tumor growth and metastasis [63]. Researchers have recently discovered that NETs play a role in the progression of tumor metastasis [64]. The original purpose of NETs was to entrap pathogens such as bacteria, fungi, protozoa, and viruses. As a result of neutrophil activation, DNA chromatin, along with histones and antibacterial proteins, is extruded from the nucleus, forming NETs [65]. They can both promote tumor metastasis and inhibit tumor growth by entrapping cancer cells and serving as adhesion substrates for cancer cells. There is compelling evidence that neutrophils can aid in remodeling the local microenvironment through NETs that further facilitate tumor cell extravasation and proliferation [63]. However, it has been shown, for instance, that NET components such as myeloperoxidase inhibit tumor growth and metastasis, especially for lung cancers [66]. Besides, with increasing numbers of NETs, more circulating tumor cells may be trapped in NETs of circulating neutrophils in blood flow toward pre-metastatic niche. An RNA-seq study reveals that lung mesenchymal stromal cells upregulate complement 3 (C3), which facilitates neutrophil recruitment and NET formation [34]. Induction and maintenance of C3 expression in lung mesenchymal stromal cells are dependent on Th2 cytokines and STAT6. There may be a promising way to prevent lung metastases from breast cancer by targeting the Th2-type cytokines-STAT6-C3-NETs axis [34]. Furthermore, it has been reported that women with early-stage ovarian tumors have NETs in their omentas [67]. Lee et al. also showed that neutrophils are attracted to the pre-metastatic omentum by tumor-derived factors, leading to the formation of NETs and the capture of cancer cells in orthotopic ovarian cancer models [67]. It has been demonstrated recently that circulating breast cancer cells can trigger neutrophils to release NETs, which led to increased migration and invasion of cancer cells to those target organs [68]. It should be noted that NET-digesting DNase I-coated nanoparticles were disclosed to suppress metastasis [68]. Besides, it has been shown by Yang et al. that NET markers are positively correlated with liver metastases in colorectal cancer patients. A combination of in vivo and ex vivo research revealed that tumor-derived factors were responsible for increased NET formation and neutrophil recruitment in pre-metastatic niche. Besides, the NETs were capable to catch disseminated colorectal cancer cells (CRC), and what is more, the generation of pro-inflammatory cytokines like IL-8, IL-6, and TNF- α was induced by them later, which can further increase the recruitment of neutrophils and NETs formation, hence promoting CRC liver metastasis [7]. In addition, it has been demonstrated that NET-embedded proteases such as neutrophil elastase (NE), myeloperoxidase (MPO), and matrix metalloproteases (MMP) cleave vascular endothelial cadherin, resulting in compromised junction integrity and therefore promoting vascular leakage [69]. A recent study has demonstrated that NETs promote angiogenesis by eliminating senescent endothelial cells and by upregulating proangiogenic factors such as VEGF [70].

## Metabolic changes of TANs

### Glycolysis of TANs

**Fig. 3:**
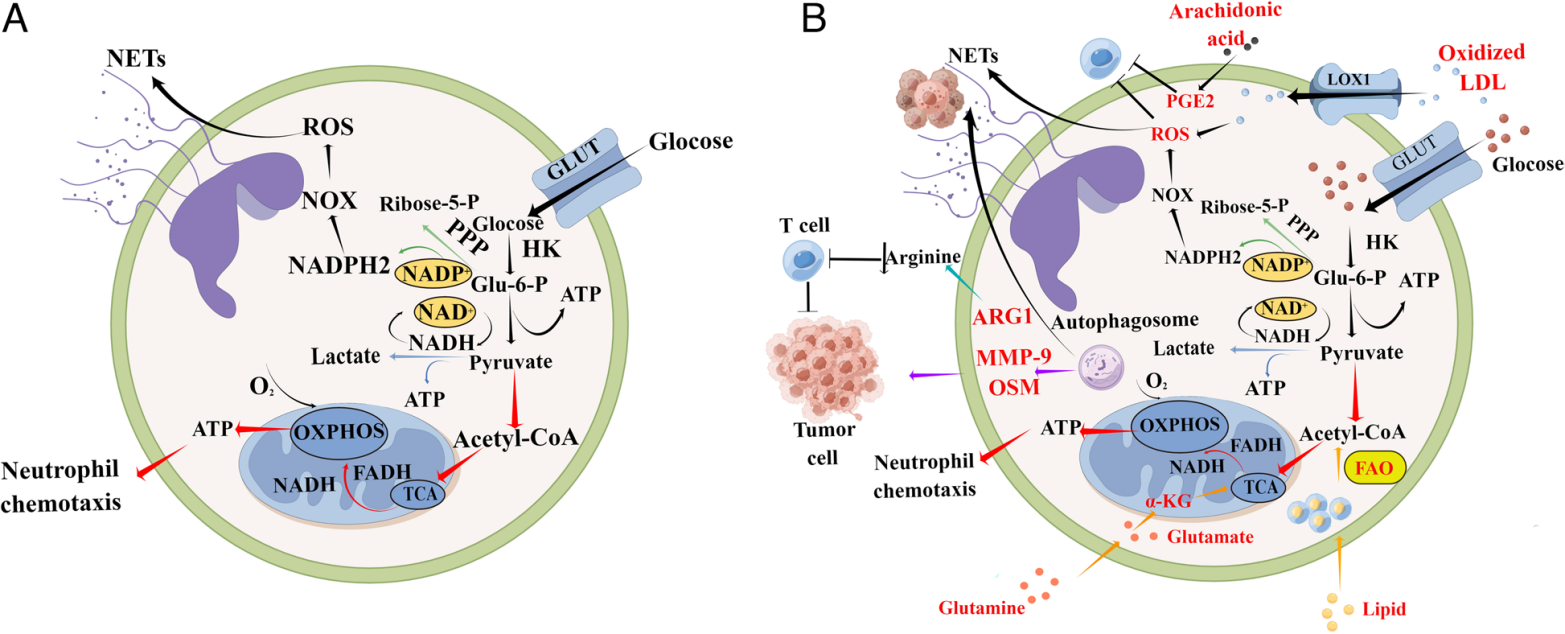
Metabolic changes of neutrophil in cancer. **A** Normal neutrophils and **B** tumor-associated neutrophils (TANs). The glycolysis process involves the degradation of glucose into pyruvate, which is converted into ATP. The metabolic process of glycolysis is fueled by NAD + produced during anaerobic conditions when pyruvate is converted into lactate. The TCA cycle produces NADPH, FADH, and ATP in the presence of oxygen by converting pyruvate to acetyl-CoA when oxygen is present. By using G6P, the PPP produces NADPH, an important mediator of NOX-dependent ROS production. The NETs formation can be triggered by ROS. TCA is carried out by acetyl-CoA produced from free fatty acids in the mitochondria. Autophagy can stimulate the production of pro-metastatic molecules such as OSM and MMP9. Reactive oxygen species, ROS; Nitrogen oxides, NOX; Pentose phosphate pathway, PPP; Hexokinase, HK; Oncostatin M, OSM; Matrix metalloprotein, MMP9; Arginase-1, ARG1; Prostaglandin E2, PGE2; Neutrophil extracellular traps, NETs; α-ketoglutarate, α -KG; Lectin-type oxidized LDL receptor 1, LOX1; Glucose Transporter, GLUT; Fatty acid oxidation, FAO; Oxidative phosphorylation, OXPHOS; Tricarboxylic acid, TCA. (Due to the inability of existing data to distinguish between N1 and N2 neutrophils, they are collectively referred to as TANs in the figure)

A neutrophil's primary source of energy during the life cycle is glucose [71, 72]. An important and fastest method of obtaining adenosine triphosphate (ATP) for neutrophils is through glycolysis, and the formation and release of NETs appears to be influenced by an increased glucose metabolism through glycolysis [73–75] (see Fig. 3A for details). In tumor-bearing animals, neutrophils utilize aerobic glycolysis similarly to cancer cells, according to transcriptomic and metabolic analysis [76]. Jiang et al. revealed that tumor-induced metabolic switches toward glycolysis and pentose phosphate pathway in tumor-infiltrating neutrophils led to the formation of NETs [77]. Patel et al. showed that in comparison with control neutrophils, TANs had higher rates of glycolysis and oxidative phosphorylation, as well as increased ATP production [78]. Besides, the activation of lactate dehydrogenase (LDH) and dimerization of pyruvate kinase type M2 (PKM2) was increased in human blood neutrophils stimulated to undergo both NOX-dependent and independent NETosis [79]. In cancer cells, both processes can contribute to the aerobic glycolysis. The identification of specific factors that stimulate neutrophil glycolysis would thus be interesting in future studies.

### Other metabolic changes of TANs

While TANs are highly dependent on glycolysis, they are also capable of circumventing it when necessary and upregulating alternate metabolic pathways such as glutaminolysis, oxidative phosphorylation (OXPHOS), and fatty acid oxidation (FAO). Thus, it is important to note that neutrophils can flexibly adapt to different microenvironments. The most significant adaptation is the metabolic alteration that has a substantial effect on cell behavior.

Hsu et al. showed that under nutrient-deprived conditions, the immature low-density neutrophils (iLDNs) remain capable of executing pro-metastatic neutrophil functions by engaging mitochondrial-dependent ATP production. In addition, without glucose, iLDNs support mitochondrially-dependent metabolism using glutamate and proline, enabling sustained NETosis, which is important for the progression of breast cancer to the liver [80]. In addition, adenosine is important for the proinflammatory effector functions of neutrophils, which facilitates neutrophil chemotaxis, phagocytosis, cytotoxicity, and cytotoxicity. However, adenosine metabolism of neutrophils was observed to be shifted to inosine metabolism in pre-metastatic niche over time. Finally, extracellular inosine molecules inhibit the production of inflammatory cytokines, which weakens antitumor immune function [81]. Rice et al. showed that oxidative mitochondrial metabolism is possible for neutrophil subsets with c-Kit + . They found that splenic neutrophils from 4T1 tumor-bearing mice are shown to have greater mitochondrial fitness via c-Kit signaling. Furthermore, peripheral blood neutrophils from cancer patients have higher mitochondrial content and oxidative phosphorylation, which is in agreement with these findings [82].

Several metabolic features have also been observed, including an upregulation of fatty acid transport protein 2 (FATP2), an increase in Arg1 levels, and an increase in nicotinamide adenine dinucleotide phosphate (NADPH) oxidase activity and activation of NADPH oxidase (NOX), which can suppress the function of T cells (see Fig. 3B for details) [83–86]. Besides, PGE2 is created by arachidonic uptake via the FATP2 transporter to inhibit T cells. By binding to lectin-type oxidized LDL receptor (LOX-1), oxidized low-density lipoprotein (LDL) can stimulate neutrophil ROS production and inhibits T cells. Neutrophils play an immunosuppressive role in T cell immunity. Blocking fatty acid oxidation influences neutrophil energy metabolism, which may synergize with T cell immunotherapy, such as anti-PD-1/PD-L1 therapy [71, 87]. This may be an effective strategy for cancer therapy. However, how to specifically target N2 neutrophils without affecting the function of N1 neutrophils is a problem to be solved.

In addition, neutrophils can upregulate autophagy in hepatocellular carcinoma to maintain mitochondrial function and survive in the tumor microenvironment. Meanwhile, autophagy stimulated pro-metastatic molecules such as OSM and MMP9 production in these cells, supporting hepatocellular carcinoma growth [88]. Besides, Peishan et al. discovered that neutrophils are induced to cumulate lipids when interacting with resident lung mesenchymal cells (MCs) in the pre-metastatic niche. These processes are triggered by lung MCs through repression of neutrophil adipotriglyceride lipase (ATGL) activity in both a prostaglandin E2-dependent and -independent manner. It is believed that the lipids in neutrophils are transported by macropinocytosis-lysosomes to metastatic tumor cells, resulting in enhanced survival and proliferation of the tumor cells. Neutrophils can serve as a source of energy to fuel the growth of breast cancer lung metastases [89].

## Summary

As a result of these findings, not only do neutrophils play a role in the growth of primary tumors, but they can also exert function in forming PRE-METASTATIC NICHE. Besides, neutrophil activity in tumors could be modulated. So, it may be possible to treat cancer in a promising manner by suppressing neutrophil pro-metastatic activity alongside traditional methods. In the aspects of metabolism, while TANs are highly dependent on glycolysis, they are also capable of circumventing it when necessary and upregulating alternate metabolic pathways such as glutaminolysis, OXPHOS, and FAO. So, targeting neutrophil metabolism pathways that activate or suppress neutrophils may serve as a useful strategy for the treatment of cancer.

## Data Availability

Not applicable.

## Abbreviations

CXCL: C-X-C motif chemokine ligand
MIP2 α: Macrophage inflammatory protein-2 alpha
IL: Interleukin
HMGB1: High-mobility group box 1
GM-CSF: Granulocyte–macrophage colony-stimulating factor
GC-SF: Granulocyte colony-stimulating factor
TNF- α: Tumor necrosis factor-alpha
LTB4: Leukotriene B4
GCP-2: Granulocyte chemotactic protein-2;
TANs: Tumor-associated neutrophils
VEGFR1: Vascular endothelial growth factor receptor 1
G-CSF: Granulocyte colony-stimulating factor
CXCR1/2: CXC chemokine receptor 1/2
CTSC: Cathepsin C
PR3: Proteinase 3
CS: Crystalline silica
ATGL: Adipotriglyceride lipase
MCs: Mesenchymal cells
NADPH: Nicotinamide adenine dinucleotide phosphate
FATP2: Fatty acid transport protein 2
ATP: Adenosine triphosphate
OXPHOS: Oxidative phosphorylation
FAO: Fatty acid oxidation
CR: Colorectal cancer cells
NETs: Neutrophil extracellular traps
ME: Mesenchymal-to-epithelial transition
LCN2: Lipocalin-2
ALOX5: Arachidonate 5-lipoxygenase
ICAM-1: Intercellular adhesion molecule 1
CTCs: Circulating tumor cells
TRAIL: TNF-related apoptosis inducing ligand
ADCC: Antibody-dependent cell-mediated cytotoxicity
MRPs: Myeloid-related proteins
HIF1α: Hypoxia-inducible factor 1α
C3: Complement component 3
LMSCs: Lung mesenchymal stromal cells
